# Sero-epidemiology of Crimean-Congo haemorrhagic fever in mixed crop-livestock farming households in Burkina Faso: a one health study

**DOI:** 10.1371/journal.pone.0347146

**Published:** 2026-05-04

**Authors:** Abdoul Kader Ilboudo, Michel Dione, Ard M. Nijhof, Martin H. Groschup, Assana Cissé, Koala Lassane, Madi Savadogo, Laibané Dieudonné Dahourou, Kristina Roesel, Roch K. Dabiré, Zekiba Tarnagda, Bernard Bett

**Affiliations:** 1 International Livestock Research Institute (ILRI), Health Program; 2 Freie Universität Berlin, Institute for Parasitology and Tropical Veterinary Medicine, Berlin, Germany; 3 Institut de Recherche en Sciences de la Santé (IRSS/CNRST), Department of Medical Biology and Public Health, Unit of Epidemic-Prone Diseases, Emerging Diseases and Zoonoses, National Influenza Reference Laboratory, Ouagadougou, Burkina Faso; 4 Veterinary Centre for Resistance Research, Freie Universität Berlin, Berlin, Germany; 5 Friedrich-Loeffler -Institut, Institute of Novel and Emerging Diseases, Insel Riems, Germany; 6 Ministry of Agriculture, Animal et Halieutic Resources, Directorate General of Veterinary Services, Directorate of Animal Health, Ouagadougou, Burkina Faso; 7 Institut des Sciences de l’Environnement et du Développement Rural (ISEDR), Université Daniel Ouezzin Coulibaly, Dédougou, Burkina Faso; 8 Department of Medical Biology and Public Health, Institut de Recherche en Sciences de la Santé (IRSS/CNRST), Bobo-Dioulasso, Burkina Faso; 9 Faculty of Veterinary Medicine, University of Liege, Fundamental and Applied Research For Animal and Health (FARAH), Liege, Belgium; 10 Department of Animal Breeding and Husbandry in the Tropics and Subtropics University of Hohenheim, South of Stuttgart, Germany; University of Ibadan Faculty of Veterinary Medicine, NIGERIA

## Abstract

**Background:**

Crimean-Congo haemorrhagic fever (CCHF) is a severe, widespread, tick-borne viral zoonotic infection. It is caused by an orthonairovirus that is transmitted by ticks. Sero-epidemiological studies in humans and livestock are valuable indicators of viral circulation and infection risk. This study aimed to investigate the seroprevalence and factors associated with CCHF virus exposure in humans and livestock in mixed crop-livestock farming households in rural Burkina Faso.

**Methods:**

A cross-sectional animal-human linked study was conducted in 149 rural households across 16 randomly selected villages in two administrative regions of Burkina Faso. Human socio-demographic, livestock biodata, and serum samples were collected from household members and their livestock (cattle, sheep, and goats). Additional ecological and climatic data were extracted from online databases and merged with the field data. Serological testing was performed on human and animal samples using the ID Screen® CCHF Double Antigen Multi-species ELISA (IDvet, Grabels, France). Descriptive statistics and multivariable multilevel analyses were used to assess factors associated with exposure of cattle and small ruminants to CCHF virus, while the Fisher’s exact test was applied to assess the risk factors for human exposure.

**Results:**

The study included 717 livestock farmers and 2,295 animals, comprising 666 cattle, 659 sheep and 970 goats. The overall CCHF virus (CCHFV) seroprevalence was 3.1% (95% CI: 1.9–4.6) in humans and 54% (95% CI: 50.2–57.7) in cattle. In small ruminants, the overall seroprevalence was 5.2% (95% CI: 4.2–6.4), with 9.1% (95% CI: 7.1–11.5) in sheep, and 2.5% (95% CI: 1.7–3.8) in goats. Farmers with inadequate livestock management-related biosecurity behaviour exhibited higher seroprevalence rates and an increased risk of CCHFV seropositivity. In cattle, seropositivity was positively associated with older age, female sex, longer grazing distances, and tick infestation. Seropositivity in small ruminants was associated with older age, being of the sheep species, and longer grazing distances. Ecological factors, including a higher aridity index in both cattle and small ruminants, and steeper slopes in cattle, were significant in univariate and multivariable analysis, respectively. The seroprevalence in both cattle and small ruminants showed significant clustering within households, with intra-cluster correlation (ICC) rates of 39% and 62%, respectively.

**Conclusion:**

This study highlighted that CCHFV is circulating among humans and their livestock in rural Burkina Faso. Individual and household-related risk factors, including socio-demographic, livestock management practices, and ecological characteristics, were identified. These findings provide valuable insights for designing tailored public health interventions towards strengthening CCHF surveillance and prevention among rural households.

## Background

Crimean-Congo haemorrhagic fever (CCHF) is a severe and often deadly viral infection. It is one of the most widespread viral zoonoses, and endemic in various countries across Eastern Europe, Africa, Asia, and the Middle East [1]. CCHF is a tick-borne infection that occurs in a broad range of vertebrates, in which it is typically asymptomatic [2]. Apart from tick bites, the virus is transmitted to humans through exposure to bodily fluids of viremic animals or patients [3]. CCHF orthonairovirus (*Orthonairovirus haemorrhagiae*) is a biosafety level 4 viral pathogen, a negative-sense RNA virus belonging to the genus *Orthonairovirus,* family *Nairoviridae* (class Bunyaviricetes order *Hareavirales*) [4]. It is characterised by a high genetic diversity, and distinct strains are distributed across endemic regions. All CCHFV genotypes share potential for human pathogenicity, however laboratory evidence shows strain-specific virulence [2,5]. The main vectors responsible for the transmission of CCHFV are ticks of the genus *Hyalomma.* The virus has also been detected in several other tick species, including ticks of the genus *Rhipicephalus, Haemaphysalis, Dermacentor, Ixodes, Amblyomma* and *Ornithodoros* [3,6], but the role of these tick species in the CCHF epidemiology and spread dynamics remains to be clarified.

Historically, the distribution of CCHF in both animals and humans was geographically restricted to specific regions where competent tick vectors were present. However, climate change has facilitated the adaptation of ticks to different environments, thus facilitating the spread of the virus to previously unaffected areas, making CCHF an emerging public health concern in many countries [7,8]. For example, no cases of CCHF were reported in Turkey in the early 2000s, but by 2020, nearly 10,000 cases had been documented [9]. Additionally, the World Health Organisation (WHO) classifies it as a priority disease for research and development due to its potential to cause major epidemics in humans. Yet, no reliable vaccines are available [10].

In sub-Saharan Africa, human cases of CCHF are still frequently reported after the first documented case occurred in the Democratic Republic of Congo in 1956 [11]. Over the past two decades, several sporadic human cases of CCHF were reported from countries such as Kenya [12], Senegal [13] and Uganda [14]. Larger outbreaks were detected in Mauritania in 2003, with 38 confirmed cases, and Sudan, with two successive epidemics in 2008 and 2009 [15,16]. Domestic animals are the amplifying hosts in the CCHF virus cycle [1], and serosurveys can be used in both humans and domestic animals to inform on CCHF virus circulation. Several studies in West Africa have shown varying seroprevalence levels depending on the regions and animal species. Low seroprevalence in cattle was found in the Democratic Republic of Congo (1.6% in Katanga province) [17], while higher seroprevalences were reported in Burkina Faso (72.2% in cattle; 14.8% in sheep) [18,19], Mali (66% in cattle) [20], Mauritania (67% in cattle, 81% in camels) [21,22], Senegal (70.3% in horses, 57.1% in cattle, 22.1% in sheep) [23], Sudan (21.3% in camels) [24].

These high seroprevalences in animals across sub-Saharan Africa reinforce the idea that the disease may be under-reported in the region [6,25]. Limited human seroprevalence studies indicate active viral circulation in countries such as Ghana [26] and Mauritania [27], with respective seroprevalence rates of 3.7% and 11.8%. However, the implementation of effective control measures and the strengthening of surveillance efforts are often hampered by the lack of robust scientific data on CCHF in many affected countries, including data on human clinical cases, prevalence in livestock and vector distribution and infection rates.

In Burkina Faso, livestock farming and trade are essential for the local economy and the livelihood of rural communities [28]. However, common rearing practices may promote the spread of the disease. Despite the recent progress in integrating human and animal disease surveillance within a One Health framework, active surveillance of potential zoonotic diseases remains limited. Therefore, reliable data on human CCHF seroprevalence in Burkina Faso are limited, with the last confirmed human case having been reported in 1984 [29]. Such data are essential for evaluating CCHF risks, particularly among rural farmers. Understanding the complex interactions between animals, humans, the environment, and tick vectors is critical to elucidating CCHF transmission dynamics. This study aims to investigate the seroprevalence and risk factors of CCHF in humans and their livestock in rural households of Burkina Faso.

## Methods

### Ethics statement

This study received ethical approval from the Burkina Faso Health Sciences Research Ethics Committee (Ref: 2022-04-081) and the Institutional Research Ethics Committee of the International Livestock Research Institute (Ref: ILRI-IREC2022-15 and ILRI-IACUC2022-14) prior to its implementation. Written informed consent was obtained from all participants or their parents/legal guardians for children (under 18 years old). All procedures adhered to relevant guidelines and regulations, consistent with the Helsinki Declaration [30]. Animals were included with the informed consent of the owner and/or the herders. Animal sampling was conducted with strict adherence to animal welfare practices.

### Study location

**Fig 1:**
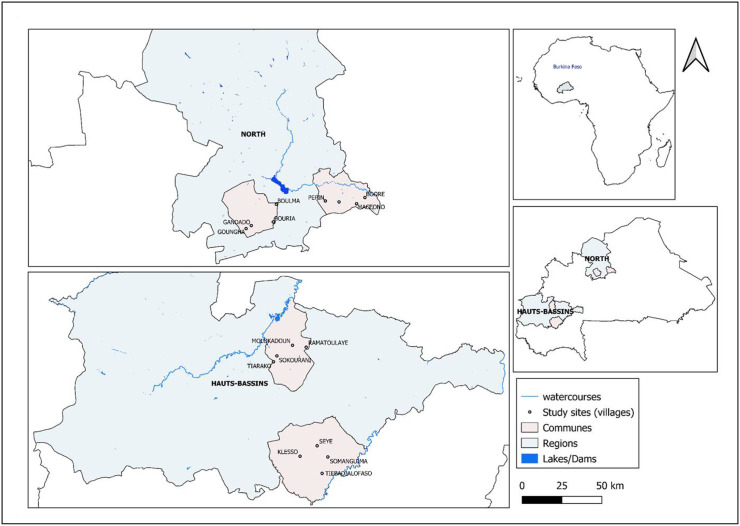
Geographic location of the study sites. Map generated by Abdoul Kader Ilboudo using QGIS version 3.36.3 and shapefiles from https://gadm.org/download_country.html, 2023.

The study framework was outlined in a previous work [31]. Briefly, Burkina Faso, a landlocked country located in West Africa with limited resources, has a human population estimated at approximately 21 million inhabitants, of which 51.7% are women. Livestock production plays a significant role in the national economy, providing essential products and services, such as food and income [32,33]. The study was conducted in two administrative regions of the country: the “Hauts-Bassins” region and the North region. These regions were selected based on their proximity to Mali, where a CCHF outbreak occurred in 2020 in the Mopti region that borders Burkina Faso [25], their involvement in intensive cross-border livestock transhumance and also based on differences in key livelihood activities, environment and livestock breeding practices between the two regions [34]. The “Hauts-Bassins” region, located in the western part of the country and covering a South-Soudanian agro-climatic zone, is characterised by mixed crop-livestock systems. In 2019, the human population was estimated at approximately 2 million inhabitants, with 51.1% being women [35]. In contrast, the North region lies in a semi-desert area and is characterised by a distinct phytogeographical zone and a predominantly extensive and semi-intensive livestock production system [28]. The human population of the North region was approximately estimated at 1,7 million, with 52.2% of females [35]. Two communes were chosen in each region based on their affluence, geographical representativeness, and accessibility regarding security issues in the country. In each commune, four villages were randomly selected from a list of all villages, ensuring alignment with the average village population size and the study’s sample size requirements, resulting in a total of sixteen villages (**Fig 1**).

### Study design and sample size calculation

A cross-sectional tick-animal-human linked study was conducted from February 6th to March 23rd, 2023.

The sample sizes were calculated for each study population (humans, cattle, sheep and goats) using the standard formula **[Formula 1]** for sample size calculation and considering assumed different prevalences from recent studies in West Africa [36]. The formula used was:


$$\text{N}=\frac{{\text{Z}}^{\text{2}}\text{p}(\text{1}-\text{p})}{{\text{e}}^{\text{2}}}*D_{eff}$$ [Formula 1]

*N is the sample size*

*Z is 1.96 at a 95% confidence level*

*p is the estimated proportion of CCHF positive individuals*

*e is the accuracy of the desired estimate (see the table below)*

*Deff is the adjustment for design effect, it is fixed at 3.7*

*The Deff is calculated using the formula:* ***Deff (DesignEffect) = 1 + ICC(K-1)***

*ICC = intra-cluster (household) correlation fixed at 0.3* [37], *K = average cluster size fixed at 10*

The assumed prevalence of CCHF among humans was estimated at 5.7% [38], and applying the above formula allows us to calculate a minimum sample size of 682 humans for the two regions, when considering a proportion of potential loss of 10%.For animals, sample sizes are evaluated according to the targeted animal species and expected prevalence (considering 10% of potential loss) with the same formula [36]. The sample size calculation was obtained per region, then added up to obtain sizes corresponding to the two regions (Table 1):

**Table 1:** Animal sample size calculation per species in Hauts-Bassins and North region, Burkina Faso, 2023.

Species	Expected prevalence	Accuracy	Calculated Sample size	Final sample size considering 10% loss
**Cattle**	60% [20]	10%	700 (350/region)	770 (385/region)
**Goats**	15% [22]	7%	740 (370/region)	814 (407/region)
**Sheep**	22,1% [23]	10%	500 (250/region)	550 (275/region)

### Participant selection

The study included males and females aged six and above from rural settings. In each of the sixteen selected villages, 8–11 households were randomly included in the study based on a list of households compiled through a preliminary census, depending on the size of the village and the number of households complying with the inclusion criteria. To be selected, a household was expected to practice crop and livestock farming and to own a minimum of ten animals (including large and small ruminants) at the time of the study or during the six months preceding the survey. A household was defined in our study as a compound with a group of individuals sharing the same agro-pastoral resources and spaces and/or the same herds of animals and organised around a head of household recognised as such. A maximum of five consenting individuals per household were then selected, based on their willingness to participate. To ensure good representativeness of each category, if the household included have children and/or women, one child and one woman were first selected from their respective lists, and then the remaining household members were randomly selected to complete the required sample, and each of them was interviewed individually. Children were interviewed with the assistance and supervision of their parents or legal guardians. Whenever possible, up to five cattle, five sheep, and ten goats aged six months and older were randomly selected from each household heard for inclusion in the study.

### Questionnaire design, data and sample collection

- **Development and quality control of data collection materials**

To ensure uniformity, the data collection instrument was initially developed in French, translated into the most commonly spoken local languages in the study regions (Dioula in “Hauts-Bassins” and Mooré in the North), and then re-translated into French and English. The questionnaire underwent pilot testing on 5% of the total sample size before being validated by the study team. Validation involved identifying and revising unclear questions post-pretest. Additionally, supervisory personnel reviewed the data daily to verify its completeness and accuracy.

- **Human, animal data and sample collection**

Socio-demographic data from selected households and their members were collected alongside specific inquiries into agro-pastoral practices, individual and collective risk factors of CCHF. The data were collected electronically on tablets using the KoboToolBox [39]. The socio-demographic data encompassed household size, the number of men, women, and children, land area cultivated, types and quantities of animals raised, and an inventory of household assets including livestock, vehicles, agricultural machinery, and motorcycles. Additionally, age, sex, and education level of each participant were recorded. Initially, the head of the household provided general household information, followed by individual interviews. Participants under 18 years old were interviewed in the presence of a parent or legal guardian. Selected animals’ biodata, including age, sex, and estimated weight by girth circumference [36–39], were also collected. Animal ages were primarily given by the owner during the interview and cross-checked by the trained veterinarian using standard dental examination techniques [40,41](S4 Appendix). The questionnaires were administered by a multidisciplinary team comprising human, animal, and community health workers through structured face-to-face interviews. They underwent training on study protocols, biosafety, sampling techniques (blood collection, tick removal), and questionnaire administration prior to fieldwork. Blood samples were collected from all consenting humans and their animals following the previously described sampling procedure. Each recruited animal was restrained by a team of trained veterinary personnel using appropriate equipment (halter, ropes and cattle nose pliers) to minimise stress and risk of injury, after which 10 mL of blood was drawn from the jugular vein into a non-heparinised tube using a G18 needle. For humans, 5 mL of blood was collected from the antecubital vein using a G22 needle, with participants seated comfortably to avoid injury. All samples, from both humans and animals, were transported separately to the local health district laboratory for serum extraction and stored at −20°C. Subsequently, samples were sent to the National Influenza Reference Laboratory in the capital city, Ouagadougou, for ELISA testing.

- **Collection of ticks from animals**

For tick collection, each animal was restrained and laid on its flank for approximately 15 minutes to facilitate the collection of all ticks attached to the opposite side. This process was repeated for the other flank so that the entire body surface of each animal was examined. Ticks were collected using forceps from typical attachment sites, e.g., the head, neck, udder, perineal region at the base of the tail, and dependent areas such as the abdomen, legs, joints, and shoulder. Collected ticks were placed into perforated 15 mL vials. Morphological identification of the ticks was conducted using the keys described by Apanaskevich *et al.,* [42,43] and Walker *et al*., [44].

- **Climatic and ecological data**

**Fig 2:**
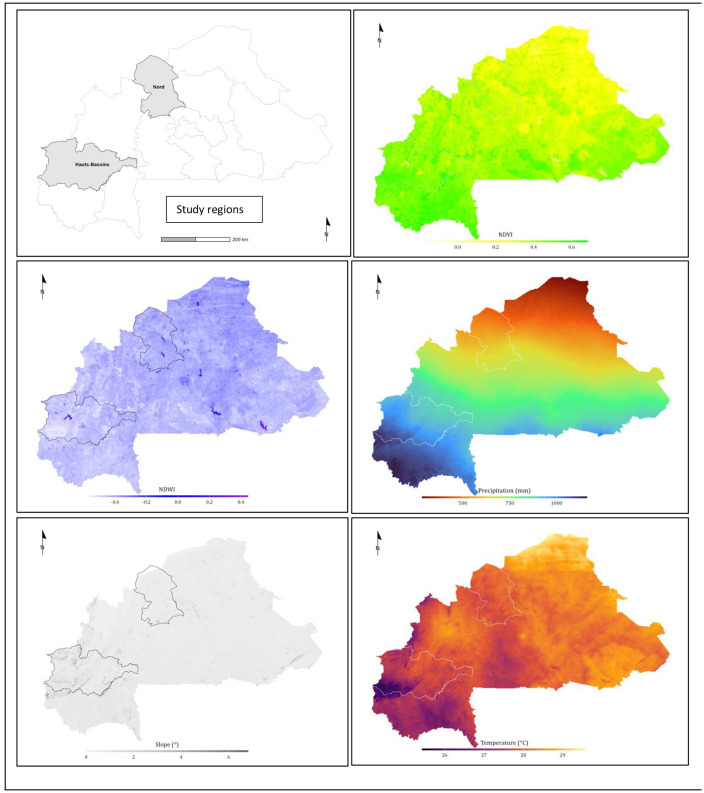
Maps showing the estimates of main geoclimatic covariates included in the CCHF risk factors analysis, (average from 1970 to 2000). *The limitations of the two regions of study are presented. The maps were designed by* **Stephen Oloo** *using QGIS version 3.36.3 and shapefiles from* https://gadm.org/download_country.html.

Climatic and environmental data were collected for each of our study sites to assess risk factors, using the GPS coordinates of the households (**Fig 2**). These data were downloaded from online sources, including: i) Climatic variables, such as annual mean precipitations and annual mean temperature obtained from WorldClim [45], and ii) geographic variables such as the digital elevation model and slope, aridity index, mean Normalized Difference Vegetation Index (NDVI), mean Modified Normalized Difference Water Index (MNDWI), and the Food and Agriculture Organisation (FAO) global landcover layers [46].

- **Serological assay**

The serological study involved analysing both human and animal serum samples to detect total antibodies specific to the CCHFV nucleocapsid protein. The ID Screen® CCHF Double Antigen Multi-species ELISA (IDvet, Grabels, France) was employed for this purpose, following the manufacturer’s established protocols. This assay is known for its high diagnostic accuracy, with a reported sensitivity ranging from 96.8% to 99.8% (95% Confidence Interval) and specificity between 99.8% and 100% (95% Confidence Interval), as validated in prior studies [47].

- **Variables definition, data management, and analysis**

The data were anonymised, cleaned and analysed using STATA 18 (StataCorp). Three outcome variables were defined based on the results of CCHF serology tests (coded 1 for positive and 0 for negative): serology test results for humans, cattle and small ruminants (combining the results from goats and sheep). The explanatory variables included household characteristics, socio-demographic characteristics of the participants, animal biodata, ticks’ carriage and annual ecological and climatic data for the study areas. Descriptive analyses were performed, including the calculation of prevalence by species and 95% confidence intervals. A series of univariate analyses was conducted between the three outcome variables and the explanatory variables. Chi-squared or Fisher’s exact tests were used to compare categorical variables.

Assuming that CCHF seroprevalence in animals may be clustered by household, we performed a two-level multilevel regression to control for the clustering effect. This group effect was assessed by testing each of the dependent variables with a null two-effect model that included random intercepts and fixed effects, with level 1 representing the individuals (n = 665 for bovines and n = 1,624 for small ruminants) and level 2 representing the household of origin (n = 140 for bovines and n = 143 for small ruminants) (S1 Appendix, S2 Appendix). We then employed a univariate multilevel regression to assess the associations between the dependent and explanatory variables. A multivariable and multilevel regression model with random intercepts and fixed effects was then initiated for the animal outcome variables (*Cattle serology results* and *small ruminant serology results*) using the stepwise forward selection method. Variables with a p-value ≤ 0.2 were progressively included to obtain the final models. The successive models were compared using AIC (Akaike Information Criterion) and BIC (Bayesian Information Criterion) to select the best fit. Post-estimation tests were conducted to assess the stability of the final models using a series of tests available in STATA 18 (StataCorp) (S3 Appendix). However, multivariable analysis was not conducted for the human serology results variable because the number of positive cases among participants (22/771) was insufficient to ensure model stability [48]. The equation of the final multilevel models for cattle and small ruminants is defined as follows [49]:


$$y_{ij=}\gamma_{\text{00 }}+\beta_{\text{1}\;}x_{ij}+\mu_{\text{0}j\;}+e_{ij}$$ [Formula 2]

*y*_ij_ is the outcome for animal i in household j

x_ij_ is the predictor at each individual animal level (cattle or small ruminant)

*β*_*1*_ is the fixed effect of the predictor,

γ_00_ is the overall intercept,

µ_0j_ is the random effect at the household level, e_i_j is the individual-level residual.

e_ij_ is the residual error term

## Results

### Household participant characteristics

A total of 717 farmers were included in 149 households across 16 villages in two regions of the country. The households visited had an average of 27.6 individuals per household (SD ± 21.3), with 52.9% housing more than 10 children. Among these households, 63.2% cultivated more than 5 hectares of land, and 92.3% owned the land they farmed, while only 14.1% possessed their own grazing areas. Additionally, 14.8% of households lacked adequate toilet facilities, and 4.2% had no access to nearby potable water (Table 2). Furthermore, 65.8% of farmers grazed their animals within 1–5 kilometers of distance from their households, while 4.7% had to go beyond 10 kilometers (Table 2). Only 20.9% of households owned a modern agricultural machine or a vehicle. The study population was relatively young, with a mean age of 32.2 years (SD ± 18), and 20.4% were under 15 years old. Farmers were predominantly male (66.4%), 67.1% had no formal education, and 61.2% were married (Table 2**).**

**Table 2:** Human and household characteristics per region, Burkina Faso, 2023.

	Human characteristics (N = 717)		
Variables	Hauts-Bassins region n (%)	North region n (%)	Total N (%)
**Age**			
<15 years	65 (19.1)	81 (21)	146 (20.4)
15-34 years	129 (37.8)	143 (38)	272 (37.9)
35 years and more	147 (43.1)	152 (40.4)	299 (41.7)
**Gender**			
Male	205 (60.1)	271 (72)	476 (66.4)
Female	136 (39.9)	105 (28)	241 (33.6)
**Education**			
None	225 (66)	256 (68.1)	481 (67.1)
Primary school	86 (25.2)	89 (23.7)	175 (24.4)
Secondary and university education	30 (8.8)	31 (8.2)	61 (8.5)
**Marital status**			
Married monogamous	84 (24.6)	93 (24.7)	177 (24.7)
Married polygamous	145 (42.5)	117 (31.1)	262 (36.5)
Single/divorced/widowed	112 (32.8)	166 (44.1)	278 (38.8)
**Communes**			
K. Vigue	166 (48.7)	–	166 (23.2)
Satiri	175 (51.3)	–	175 (24.4)
Yako	–	197 (52.4)	197 (27.5)
Tema-Bokin	–	179 (47.6)	179 (25)
Number of households included	73 (49)	76 (51)	149 (100)
**Household size**			
2-15 persons	141 (41.3)	52 (13.8)	193 (26.9)
16-35 persons	173 (50.7)	196 (52.1)	369 (51.5)
36 persons and more	27 (7.9)	128 (34.0)	155 (21.6)
**Number of men in the household**			
1-5 men	192 (56.3)	142 (37.8)	334 (46.6)
6 men and more	149 (43.7)	234 (62.2)	383 (53.4)
**Number of women in the household**			
1-5 women	205 (60.1)	112 (29.8)	317 (44.2)
6 women and more	136 (39.9)	264 (70.2)	400 (55.8)
**Number of children in the household**			
1-9 children	226 (66.3)	112 (29.8)	338 (47.1)
10 children and more	115 (33.7)	264 (70.2)	379 (52.9)
**Farming surface**			
1-4 hectares	101 (29.6)	163 (43.4)	264 (36.8)
5 hectares and more	240 (70.4)	213 (56.6)	453 (63.2)
**Household owns the farmland**			
Yes	301 (88.3)	361 (96)	662 (92.3)
No	40 (11.7)	15 (4)	55 (7.7)
**Households own the grazing areas**			
Yes	119 (34.9)	356 (94.7)	13 (14.1)
No	222 (65.1)	356 (94.7)	578 (80.6)
**Minimum distance of livestock grazing area**			
< 1 km	97 (28.4)	89 (23.7)	186 (25.9)
1-5 km	186 (54.5)	279 (74.2)	465 (64.8)
6-10 km	27 (7.92)	5 (1.3)	32 (4.5)
>10 km	31 (9.1)	3 (0.8)	34 (4.7)
**Household has access to water**			
Yes	341 (100)	346 (92)	687 (95.8)
No	0 (0)	30 (8)	30 (4.2)
**Main source of water**			
Running water	0 (0)	21 (5.6)	21 (2.9)
Common borehole	252 (73.9)	278 (73.9)	530 (73.9)
Individual borehole	9 (2.6)	10 (2.7)	19 (2.6)
well	80 (23.5)	67 (17.8)	147 (20.5)
**Main source of power**			
National grid	7 (2.3)	15 (4.8)	22 (3.5)
Own sources	304 (97.7)	297 (95.2)	601 (96.5)
**Television in the household**			
Yes	176 (51.6)	71 (18.9)	247 (34.4)
No	165 (48.4)	305 (81.1)	470 (65.6)
**Radio in the household**			
Yes	261 (7605)	323 (85.9)	584 (81.5)
No	80 (23.5)	53 (14.1)	133 (18.5)
**Toilets in the household**			
Yes	295 (86.5)	316 (84)	611 (85.2)
No	46 (13.5)	60 (16.0)	106 (14.8)
**Agricultural machine/ car in the household**			
Yes	75 (22)	75 (19.9)	150 (20.9)
No	266 (78)	301 (80.1)	567 (79.1)
**Motorcycle in the household**			
Yes	303 (88.9)	316 (84)	619 (86.3)
No	38 (11.1)	60 (16)	98 (13.7)
**Main fuel for used cooking**			
wood	270 (76.2)	329 (87.5)	599 (83.5)
charcoal	71 (20.8)	47 (12.5)	118 (18.5)
**Type of breeding**			
Extensive	330 (96.8)	376 (100)	706 (98.5)
Semi-extensive	11 (3.2)	0 (0)	11 (1.5)
**Main purpose of breeding**			
fattening (selling)	336 (98.5)	376 (100)	712 (99.3)
dairy production	5 (1.5)	0.0 (0.0)	5 (0.7)

### Livestock characteristics

A total of 2,295 animals (cattle: 666, sheep: 659, goats: 970), in 149 households across 16 villages in two regions of the country, were included in the study. Females were predominant among the three domestic animal species included in the study: 56.3% of all cattle, 69.2% of all sheep, and 74.9% of all goats. The mean ages were 48 months (SD ± 30.1) for cattle, 33.4 months (SD ± 18.4) for sheep, and 31 months (SD ± 18.4) for goats. The estimated mean weights were 153.3 kg (SD ± 55.4) for cattle, 26.6 kg (SD ± 8.2) for sheep, and 25.1 kg (SD ± 8.8) for goats. Nearly all animals (97.8%) were part of mixed-species herds, and 98.4% were raised under extensive farming conditions. Additionally, 98% of the herds were in contact with other herds.

### Tick abundance and diversity

A total of 1,517 adult ticks were collected. Tick infestations were observed in 42.3% of cattle, with a mean burden of 2.2 ticks per animal. In contrast, only 2.3% of sheep and 0.3% of goats were infested (**Table 3). Table 4** summarises the diversity and characteristics of the collected ticks. In total, ten species belonging to three genera were identified: *Amblyomma*, *Hyalomma*, and *Rhipicephalus* (including the subgenus *Boophilus*). Within the *Amblyomma* genus, only *Amblyomma variegatum* was detected. The *Hyalomma* genus was represented by four species, of which *Hyalomma truncatum* (41%) and *Hyalomma rufipes* (36.5%) were the most prevalent. Similarly, five species were identified within the *Rhipicephalus* genus, including the invasive *Rhipicephalus (Boophilus) microplus* alongside native species such as *R. (B.) decoloratus* and *R. (B.) geigyi*.

**Table 3:** Characteristics of the animal population in the Hauts-Bassins and North regions, Burkina Faso, 2023.

	*Species (N = 2295)*			
Variables	Cattle (n = 666)	Sheep (n = 659)	Goats(n = 970)	Total
**Regions n(%)**				
Hauts-Bassins	320 (48.1)	290 (44)	292 (30.1)	902 (39.3)
Nord	346 (51.9)	369 (56)	678 (69.9)	1393 (60.7)
**Sex n(%)**				
Male	291 (43.7)	203 (30.8)	243 (25.1)	737 (32.1)
Female	375 (56.3)	456 (69.2)	727 (74.9)	1558 (67.8)
Age in month (mean ±SD*)	48 (±30.1)	33.4 (±18.4)	31 (±18.4)	36.6 (±23.5)
Weight (mean±SD)	153.3 (±55.4)	26.6 (±8.2)	25.1 (±8.8)	62.7 (±65.5)
**Type of heard n(%)**				
Mixed herds	648 (97.3)	641 (97.3)	955 (98.5)	2244 (97.8)
Unique species herds	18 (2.7)	18 (2.7)	15 (1.5)	51 (2.2)
Animal owner’s age (mean±SD)	51.2 (±13.4)	51 (±12.6)	51.6 (±13.4)	51.3 (±13.2)
**Type of breeding n(%)**				
Extensive	651 (97.8)	644 (97.7)	963 (99.3)	2258 (98.4)
Semi-extensive	15 (2.2)	15 (2.3)	7 (0.7)	37 (1.6)
**Sickness observed on the animal during the last 6 months n(%)**				
Yes	359 (53.9)	337 (51.1)	548 (56.5)	1244 (54.2)
No	307 (46.1)	322 (48.9)	422 (43.5)	1051 (45.8)
**Contact with other herds n(%)**				
Yes	654 (98.2)	647 (98.2)	947 (97.6)	2248 (98)
No	12 (2.8)	12 (1.8)	23 (2.4)	47 (2)
**Presence of ticks n(%)**				
Yes	282 (42.3)	15 (2.3)	3 (0.3)	300 (13.1)
No	384 (57.7)	644 (97.7)	967 (99.7)	1995 (86.9)
Number of ticks collected (mean±SD)	2.2 (±4.4)	0.1 (±1.1)	0.01 (0.06)	0.6 (±2.6)

*Standard deviation

**Table 4:** Specific distribution of ticks by study sites (Regions/communes), Burkina Faso, 2023.

	Regions	Hauts Bassins		North		
	Communes	Satiri	Karangasso-vigué	Yako	Tema-Bokin	Total n(%)
Genus	Species					
*Amblyomma*	*Amblyomma variegatum*	18	5	44	34	**101 (6.7)**
*Hyalomma*	*Hyalomma spp.*	1	0	0	0	**1 (0.1)**
*Hyalomma*	*Hyalomma rufipes*	379	83	56	36	**554 (36.6)**
*Hyalomma*	*Hyalomma truncatum*	391	65	108	56	**620 (40.9)**
*Hyalomma*	*Hyalomma impressum*	37	0	0	0	**37 (2.4)**
*Rhipicephalus*	*Rhipicephalus (Boophilus) spp.*	47	57	7	0	**111 (7.3)**
*Rhipicephalus*	*Rhipicephalus (Boophilus) decoloratus*	8	0	1	3	**12 (0.8)**
*Rhipicephalus*	*Rhipicephalus (Boophilus) annulatus*	5	0	0	0	**5 (0.3)**
*Rhipicephalus*	*Rhipicephalus (Boophilus) microplus*	27	10	0	0	**37 (2.4)**
*Rhipicephalus*	*Rhipicephalus (Boophilus) geigyi*	6	17	0	0	**23 (1.5)**
*Rhipicephalus*	*Rhipicephalus sanguineus* sensu lato	0	13	1	0	**14 (0.9)**
	**Total (communes) n (%)**	**921 (60.7)**	**250 (16.5)**	**217 (14.3)**	**129 (8.5)**	**1517 (100)**
	**Total (regions) n (%)**	**1171 (77.2)**		**346 (22.8)**		

### CCHF seroprevalence and associated factors among humans

The overall seroprevalence of CCHF antibodies among farmers was 3.1% (95% CI: 1.9–4.6). Seroprevalence was higher in females (3.6%) compared to males (2.1%) and varied by age group: 4.7% in individuals aged ≥35 years, 2.6% in those aged 15–34 years, and 0.7% in those under 15 years. Seroprevalence also differed by education level, with no cases detected among individuals with secondary or university education, 1.1% among those with primary education, and 4.2% among those with no formal education. Overall, seroprevalence was higher among individuals engaging in high-risk practices, such as a history of exposure to animal fluids, assisting livestock parturition with bare hands, having a history of tick bites and their removal/crushing with bare hands, and participating in animal slaughtering. The duration of contact with livestock was significantly related to CCHF seropositivity, with those in higher categories of contact (6–12 hours/day and >12 hours/day) showing higher seroprevalence (p = 0.014). Other risk factors associated with CCHF seropositivity included cleaning livestock pens (p = 0.039) and handling carcasses without protection (p = 0.016) (Table 5**).**

**Table 5:** CCHF seroprevalence and associated factors among the human study population in Hauts-Bassins and North regions, Burkina Faso, 2023.

	*Seroprevalence human (N = 717)*		Fischer Exact test (F)
Variables	n/N	Prevalence(%) CI(95%)	p
**Total**	22/717	3.1 (1.9-4.6)	
**Age**			0.065
<15 years	1/146	0.7 (0.2-3.7)	
15-34 years	7/272	2.6 (1-5.2)	
35 Years and more	14/299	4.7 (2.6-7.7)	
**Region**			0.542
Hauts-Bassins	12/341	3.5 (2-6.1)	
North	10/376	2.7 (1.4-4.9)	
**Gender**			0.361
Male	5/240	2.1 (0.6-4.8)	
Female	17/477	3.6 (2.1-5.6)	
**Education**			0.063
None	20/481	4.2 (2.5-6.3)	
Primary school	2/175	1.1 (1-3)	
Secondary and university education	0/61	0	
**Marital status**			0.059
Married monogamous	5/177	2.8 (0.9-6.5)	
Married polygamous	13/262	4.9 (2.7-8.3)	
Single/divorced/widowed	4/278	1.4 (0.4-3.6)	
**Duration of contact with livestock on typical day**			0.014*
<1H/day	10/268	3.7 (1.8-6.7)	
1-6H/ day	4/325	1.2 (0.3-3.1)	
7-12H/day	3/44	6.8 (1.4-18.6)	
>12H/ day	5/80	6.2 (2.1-14)	
Regular use of PPE**during high-risk contact			0.222
Always	0/9	0	
Often	0/12	0	
Rarely	3/235	1.3 (0.3-3.7)	
Never	19/461	3.1 (2.4-6.4)	
**Clean animals’ pens**			0.039*
No	9/159	5.7 (2.6-10.5)	
Yes	13/558	2.3 (1.2-3.9)	
**Give health care to animals**			0.556
No	20/606	3.3 (2-5)	
Yes	2/111	1.8 (0.2-6.3)	
**Touch animal blood with bare hands**			0.190
No	15/568	2.6 (1.5-4.3)	
Yes	7/149	4.7 (1.9-9.4)	
**Assist animal during parturition**			0.08
No	16/614	2.6 (1.5-4.3)	
Yes	6/103	5.8 (2.2-12.2)	
**Slaughter animals**			0.067
No	15/593	2.5 (1.4-4.1)	
Yes	7/127	5.6 (2.3-11.3)	
**Touch animal carcass without protection**			0.016*
No	12/557	2.1 (1.1-3.7)	
Yes	10/160	6.3 (3-11.2)	
**Recent history of tick bite**			0.505
No	7/293	2.4 (0.9-4.9)	
Yes	14/409	3.4 (1.9-5.7)	
**Remove/crush ticks with hands during biting**			0.373
No	6/260	2.3 (0.9-4.9)	
Yes	16/457	3.5 (2-5.6)	

*P < 0.05;

### CCHF seroprevalence and associated factors among cattle

The overall seroprevalence of CCHF in cattle was 54% (95% CI: 50.2–57.7) and varied significantly across several factors. Regional differences were observed, with a higher prevalence in the “Hauts-Bassins” region compared to the North (p = 0.004). Seroprevalence was significantly associated with sex, with male cattle showing lower odds of positivity than females (p = 0.043). Age was also a strong predictor in both univariable and multivariable analyses, with older cattle (≥ 60 months) having the highest seroprevalence (p < 0.001). Cattle with higher estimated weights had a significantly higher prevalence, with animals weighing 150–199 kg or ≥200 kg having increased odds of seropositivity (p < 0.001). The presence of ticks was also significantly associated with seropositivity (p = 0.007), and cattle with more than five ticks collected had a significantly higher seroprevalence (p = 0.006). Cattle grazing 1–5 km away from farms had significantly higher seroprevalence compared to those grazing nearby (p = 0.024) in univariate analysis. Moreover, geoclimatic factors, including slope, aridity index, mean temperature, and precipitation, exhibited a significant association with CCHF seropositivity in cattle in the univariate analysis. In the adjusted model, living in steeper slopes remained significantly associated with CCHF seropositivity (p = 0.007) (Table 6).

**Table 6:** Seroprevalence of CCHF in cattle and associated factors in the multivariable multilevel regression analysis in Hauts-Bassins and North regions, Burkina Faso, 2023.

	*Seroprevalence in cattle (N = 666)*				
Variables	Prevalence (CI95%)	Crude OR (CI95%)	p-value	Adj Coef^1^ (CI95%)	p-value
**Regions**					
Hauts-Bassins	69.1 (63.8-73.9)	4.6 (2.6-8.1)	<0.001***	−0.9 (−1.5;-0.3)	0.004**
North	39.9 (34.9-45.3)	1 (base)	–	0 (base)	–
**Sex**					
Male	52.2 (46.5-57.9)	1 (base)	–	0.5 (0.01-1)	0.043*
Female	55.3 (50.3-60.3)	1.1 (0.7-1.6)	0.787	0 (base)	–
**Age in months**				0.02 (0.01-0.03)	<0.001***
6-12 months	26.1 (18.1-36)	1 (base)	–		
13-24 months	29.6 (21.9-38.5)	1.2 (0.6-2.8)	0.629		
25-59 months	57.1 (49.8-64)	6.2 (2.8-13.5)	<0.001***		
60 months and more	71.5 (65.7-76.6)	15.4 (7-33.9)	<0.001***		
**Weight**				0.01 (0.002-0.01)	0.004**
<100 kg	23.8 (16.4-33)	1 (base)	–		
100-149 kg	51.9 (45.5-58.3)	2.4 (1.2-4.8)	0.018**		
150-199 kg	67 (60-73.5)	7 (3.3-14.4)	<0.001***		
200 kg and more	61.6 (53.5-69)	6.8 (3.2-14.7)	<0.001***		
**Type of heard**				–	–
Mixed herds	53.9 (50.1-57.7)	1 (base)	–		
Unique species herds	55.5 (33-76)	1.1 (0.2-7.2)	0.890		
**Animal owner’s age**					
<40 years	65.1 (57-72.4)	1 (base)	–	–	–
40-59 years	52 (46.5-57.4)	0.4 (0.2-0.9)	0.036**		
60 years and more	49 (42-56)	0.4 (0.1-0.9)	0.025**		
**Type of breeding**				–	–
Extensive	53.4 (49.5-50.5)	1 (base)	–		
Semi-extensive	80 (53-57.2)	6 (0.6-64.1)	0.136		
**Presence of tick on the animal**				–	–
Yes	61.3 (55.5-66.9)	1.8 (1.2-2.8)	0.007**		
No	48.5 (43.5-53.6)	1 (base)	–		
**Number of ticks collected on the animal**				0.1 (0.02-0.2)	0.024*
None	50.9 (45.9-55.8)	1 (base)	–		
1-5 ticks	46.5 (42.5-56.5)	1 (0.6-1.6)	0.750		
>5 ticks	78.6 (68.5-86.1)	3.7 (1.7-7.9)	0.001**		
**Total herd size (small+large ruminants)**				–	–
< 20 heads	61.3 (51.7-70.1)	1 (base)	–		
21-50 heads	51.3 (46-56.7)	0.5 (0.2-1.3)	0.195		
More than 50 heads	54.4 (48-60.9)	0.5 (0.3-1.6)	0.365		
**Estimated cultivated area**				0.03 (0.0003-1)	0.043*
1-4 hectares	47.3 (41.1-53.6)	1 (base)	–		
5 hectares and more	57 (52.2-61.7)	1.6 (0.9-3.2)	0.101		
**Estimated distance of grazing area**				–	–
None (around farm)	50.6 (46.5-54.7)	1 (base)	–		
1-5 km	80 (64.5-90)	6.3 (1.6-24.8)	0.009**		
6 km and more	68.3 (55.5-78.8)	2.7 (0.9-7.8)	0.06		
**Animal in contact with transhuman herds**				–	–
No	36.3 (14.3-66.2)	1 (base)	–		
Yes	54.6 (50.7-58.4)	1.6 (0.2-12.8)	0.620		
**Household size**				–	–
2-15 individual	61 (53.5-67.8)	2.3 (1-5.5)	0.058		
16-35 individuals	52.4 (47-57.7)	1.4 (0.6-3)	0.386		
36 and more	46.5 (38.5-54.7)	1 (base)	–		
**Household members use adequate toilettes**				–	–
No	45.9 (36.3-55.8)	1 (base)	–		
Yes	54.7 (50.6-58.8)	1.6 (0.7-3.7)	0.296		
**Human CCHF seropositive in the household**				–	–
No	53.3 (49.2-57.4)	1 (base)	--		
Yes	60.7 (49.9-70.5)	1.4 (0.6-3.5)	0.461		
**Variables**	**Mean (±SD)**	**Crude Coef(CI95%)**	**p-value**	**Adj Coef**	**p-value**
Slope	0.4 (0.21)	2.6 (1.2-3)	<0.001***	2 (0.6-3.5)	0.007**
Mean NDVI	0.1 (0.0.04)	5.5 (−1.8-12.7)	0.141	–	–
Mean MNDWI	−0.3 (0.03)	0.7 (−10.4-11.9)	0.894	–	–
Aridity	0.3 (0.09)	7.6 (4.7-10.5)	<0.001***	–	–
Annual Mean Temperature	3012.2 (1.88)	−0.4 (−0.5;-0.2)	<0.001***	–	–
Annual Mean Precipitation	8037.4(1352.2)	0.001 (0.0003-0.0007)	<0.001***	–	–

*P<0.05; **P<0.01; ***P<0.001; 1 = adjusted coefficient in the multivariable analysis

### CCHF seroprevalence and risk factors among small ruminants

The overall seroprevalence of CCHF in small ruminants was 5.2% (95% CI: 4.2–6.4), 9.1% (95% CI: 7.1–11.5) in sheep, and 2.5% (95% CI: 1.7–3.8) in goats. The seroprevalence was significantly higher in the “Hauts-Bassins” region compared to the North (p < 0.001). A significant difference (p = 0.007) was observed in seroprevalences between sheep and goats, with rates of 9.1% (95% CI: 7.1–11.5) in sheep and 2.5% (95% CI: 1.7–3.8) in goats. Older sheep and goats (≥ 25 months) had a significantly higher seroprevalence than younger ones (p = 0.003). Higher grazing distance and steeper slopes in grazing areas were associated with increased seroprevalence in an univariable analysis. Higher vegetation density (NDVI) was also positively correlated with seroprevalence in univariate analysis (p = 0.020) (Table 7).

**Table 7:** Seroprevalence of CCHF in small ruminants* and associated factors in the multivariable multilevel regression analysis, Burkina Faso, 2023.

	*Seroprevalence in small ruminants (N = 1614)*				
Variables	Prevalence (CI95%)	Crude OR (CI95%)	p-value	Adj coef^1^ (CI95%)	p-value
**Regions**					
Hauts-Bassins	12.5 (10-15.4)	20.1 (7.2-52.1)	<0.001***	2.7 (1.8-3.6)	<0.001***
North	1.2 (0.7-2.1)	1 (base)	–	0 (base)	
**Sex**				–	–
Male	8.5 (6.3-11.5)	1.4 (0.8-2.5)	–	–	–
Female	4 (3-5.3)	1 (base)	0.219	–	–
**Species**					
Sheep	9.1 (7.1-11.5)	3.1 (1.7-5.5)	<0.001***	0.8 (0.2-1.4)	0.007**
Goats	2.6 (1.7-3.8)	1 (base)	–	0 (base)	
**Age in months**				0.03 (0.02-0.05)	<0.001***
6-12 months	1.7 (0.8-3.8)	1 (base)	–		
13-24 months	3.7 (2.3-5.9)	2 (0.7-5.5)	0.197	–	–
≥25 months	7.2 (5.6-9.2)	4.1 (1.6-10.5)	0.003**	–	–
**Weight**				–	–
<30 kg	5.1 (3.9-6.6)	1 (base)	–	–	–
30-39 kg	5.2 (3.5-7.7)	1.7 (0.9-3.2)	0.094	–	–
40-and more	6.5 (3.3-12.6)	2.4 (0.9-6.5)	0.084	–	–
**Type of herd**				–	–
Mixed herds	5 (4.1-6.2)	1 (base)	–	–	–
Unique species herds	15.2 (6.4-31.6)	4.4 (0.2-81.5)	0.315	–	–
**Animal owner’s age**				–	–
<40 years	6.6 (4.3-10)	1 (base)	–	–	–
40-59 years	5.2 (3.9-7)	0.6 (0.2-2.3)	0.472	–	–
60 years and more	4.4 (2.9-6.6)	0.5 (0.5-2.2)	0.361	–	–
**Type of breeding**				–	–
Extensive	5.1 (4.1-6.3)	1 (base)	–	–	–
Semi-extensive	13.6 (4.4-34.8)	5.4 (0.2-127.7)	0.290	–	–
**Presence of tick on the animal**				–	–
Yes	16.6 (5.4-40.9)	2.2 (0.4-10.5)	0.338	–	–
No	5.1 (4.1-6.3)	1 (base)	–	–	–
**Number of ticks collected on the animal**				–	–
None	10.2 (8-12.5)	1 (base)	–	–	–
1-5 ticks	21.4 (7.1-49.5)	2.2 (0.5-10.3)	0.330	–	–
>5 ticks	0	0	–	–	–
**Total herd size (small+large ruminants)**				–	–
< 20 heads	7.2 (4.3-11.8)	1 (base)	–	–	–
21-50 heads	4 (2.9-5.6)	0.4 (0.1-8)	0.246	–	–
More than 50 heads	6.3 (4.6-8.6)	1.1 (0.2-4.8)	0.919	–	–
**Estimated cultivated area**				–	–
1-4 hectares	4.7 (3.2-6.7)	1 (base)	–	–	–
5 hectares and more	5.5 (4.2-7)	1.3 (0.4-4.2)	0.611	–	–
**Estimated distance of grazing area**				–	–
None (around farm)	4.1 (3.2-5.3)	1 (base)	–	–	–
1-5 km	15.3 (8.7-25.5)	7.6 (1.3-43.3)	0.023*	–	–
6 km and more	11.7 (7.3-18.3)	7.2 (1.5-34.8)	0.015*	–	–
**Household size**				–	–
2-15 individual	7.6 (5.3-10.7)	1 (base)	–	–	–
16-35 individuals	4.8 (3.5-6.5)	0.5 (0.1-1.7)	0.291	–	–
36 and more	3.7 (2.2-6.1)	0.4 (0.1-1.9)	0.292	–	–
**Household members used adequate toilettes**				–	–
No	8 (5-12.4)	1 (base)	–	–	–
Yes	4.7 (3.8-6)	0.5 (0.1-2.2)	0.368	–	–
**Human CCHF seropositive in the household**				–	–
No	5.1 (4.1-6.4)	1 (base)	–	–	–
Yes	6 (3.4-10.3)	1.7 (0.4-2.9)	0.484	–	–
**Variables**	**Mean (±SD)**	**Crude Coef(CI95%)**	**p-value**	**Adj Coef**	**p-value**
**Slope**	0.4 (0.21)	2.3 (0.2-4.5)	0.035*	–	–
**Mean NDVI**	0.1 (0.0.04)	14.2 (2.2-26.1)	0.020**	5.6 (−2.9-14.1)	0.201
**Mean MNDWI**	−0.3 (0.03)	14.4 (−4.6-33.5)	0.139	–	–
**Aridity**	0.3 (0.09)	15.2 (10.4-20.1)	<0.001***	–	–
**Annual Mean Temperature**	3012.2 (1.88)	−0.7 (−1.1;-0.3)	0.001**	–	–
**Annual Mean Precipitation**	8037.4 (1352.2)	0.001 (0.0007-0.001)	<0.001***	–	**–**

*P < 0.05; **P < 0.01; ***P < 0.001; 1: adjusted coefficient in the multivariable analysis

### Seroprevalence clustering effect determination

A null two-level mixed-effects logistic regression model was used to estimate the level of clustering effect for both cattle and small ruminants seroprevalence. The Likelihood Ratio tests performed to compare the mixed model to a standard logistic model were significant for the two models (p < 0.001), and so, there was strong evidence that the between-household variance for CCHF seroprevalence among cattle and small ruminants was non-zero. The intra-cluster correlations (ICC) were 0.39 and 0.61, respectively, for cattle and small ruminants. This indicates that 39% and 61% of the total variance in cattle and small ruminant seropositivity, respectively, was attributable to differences between households, rather than between individual animals (S1 Appendix).

## Discussion

Our study aimed to determine the seroprevalence and risk factors of CCHF among human populations and their livestock in rural mixed crop-livestock farming households using a One Health approach. For this purpose, a human-animal-ecological-tick-linked study was conducted in two regions of Burkina Faso. This approach allowed us to assess seroprevalence across different targeted animal species and identify both individual and collective risk factors at intra- and inter-group levels. Our findings confirm the circulation of CCHF orthonairovirus among farmers and their livestock in mixed crop-livestock farming households. The study detected human seropositive cases and revealed a relatively high seroprevalence in cattle, whereas small ruminants exhibited a lower prevalence. Moreover, multilevel statistical analyses indicated that CCHF seropositivity is clustered at the household level for both cattle and small ruminants. The models demonstrate that collective factors, such as household socio-economic characteristics and ecological factors, primarily drive CCHF seroprevalence in domestic animals. Additionally, individual animal characteristics, human behavioural factors, and ticks’ infestation level were identified as key determinants of CCHF seroprevalence-associated risk.

To our knowledge, this study is the first to assess the seroprevalence of CCHF in humans and their owned livestock in Burkina Faso. The last reported human case dates back to 1984 and involved an individual presenting haemorrhagic symptoms [29]. Combined weaknesses in case detection, notification, and laboratory diagnostic capacity, together with the possibility that locally circulating CCHFV strains often cause mild or subclinical infections, may explain the absence of reported clinically active cases since the first description of CCHF in the country [50,51]. In this work, we found a seroprevalence of 3.1% among farmers practising mixed crop-livestock farming. These individuals are regularly exposed to tick bites and direct contact with the bodily fluids of animals due to their occupational activities [52,53].

Higher seroprevalences have been reported in high-risk occupational groups and febrile patients in other countries: 5.7% in abattoir workers in Ghana [38], 11.8% in a retrospective study among slaughterhouses workers in Mauritania (2020–2021) [27], 10.6% among febrile patients in Nigeria (2010–2014) [54], 4.4% among Pygmy populations in Cameroon (2005–2012) [55], 7.2% in rural farmers in Kenya [56], and up to 25.6% in febrile patients in Kenya [57]. A seroprevalence close to our findings (3.8%) was also reported among farmers in South Africa [58]. Notably, these studies were conducted mainly in symptomatic patients or high-risk occupational workers, and often in countries that had previously experienced CCHF outbreaks, which is not the case in Burkina Faso.

Seroprevalence was higher among individuals engaged in risky biosafety practices, and was significantly associated with the amount of time spent in contact with animals [53]. Contact with animal fluids under poor biosafety conditions is a well-established risk factor for both seropositivity and clinical cases of CCHF [2,3,5,59]. Consequently, categories such as livestock professionals, livestock keepers, herders, abattoir workers, meat traders, as well as meat consumers may be at high risk of infection to CCHF [38,58,60].

According to previous studies, human seroconversion is rapid and may persist for up to five years [61–63]. Moreover, certain sociocultural and livestock management practices, such as home slaughter and extensive grazing systems, have been epidemiologically linked to CCHF spread [9,64]. However, it should be noted that seroprevalence data reflect exposure risk and do not necessarily correlate with the risk of clinical CCHF cases. This potential discrepancy may be explained by limited knowledge regarding the relationship between viral infection and symptom development, which may be virus-strain dependent [65]. Additionally, the predominance of subclinical forms among infected cases and limited diagnostic capacity may contribute to the “silent circulation” of the virus in human populations [3].

In animals, we found a high seroprevalence among cattle (54%), consistent with previous findings in Burkina Faso [19] and other West African countries such as Mali (66%) [20] and Mauritania (67%) [21]. In contrast, seroprevalence was substantially lower among small ruminants (9.1% in sheep and 2.5% in goats), which aligns with the known ecological role of vertebrate hosts in the *Hyalomma* life cycle. In many *Hyalomma* ticks, immature stages preferentially parasitise small mammals such as rodents and lagomorphs, while adult ticks predominantly feed on large ruminants, particularly cattle [6,44,65,66]. Additionally, the larger size of cattle compared to sheep and goats likely increases their susceptibility to tick infestation, thereby elevating their risk of infection. Seroprevalence in cattle was significantly higher in animals from which more than five ticks were collected, as shown in both univariate and multivariable analyses. While the timing of tick collection does not reflect the moment of infection, it might serve as a proxy for a history of more frequent or intense tick infestations and thus a higher risk of becoming exposed to CCHFV. A significant difference in seroprevalence was also observed between sheep and goats, with sheep showing a higher rate of exposure. This is consistent with findings from similar studies [27,67], suggesting potential species-specific differences in susceptibility or tick-host interactions and species-specific husbandry practices.

Other risk factors for CCHF seropositivity in animals included biological characteristics such as age (in both cattle and small ruminants), sex (cattle), and estimated weight (cattle). The association with age is likely related to cumulative exposure to infected ticks over time, particularly given that antibody responses can persist for several years [23]. Similar findings have been reported in sub-Saharan Africa for cattle and other domestic animals [22,27,58]. The associations with sex and weight may partly reflect age-related factors and may also be linked to husbandry practices that increase exposure of female cattle to infection [68]. Indeed, in local breeding practices, female animals are kept on farms for as long as possible to ensure reproduction and herd renewal, while adult males are generally sold for income. Extensive livestock production also plays an important role by facilitating contact between herds, including those from potentially endemic areas [27].

Our study also investigates the diversity and characteristics of ticks collected on livestock across the two regions and four communes. The greatest diversity of ticks was observed in the Hauts-Bassins region, in the communes of Satiri and Karangasso Vigué. This aligns with tick densities observed in these areas, which contrasts with the lower densities reported in communes located in the North region. The variation in tick populations across these regions suggests that ecological factors, such as vegetation, climate, and livestock management practices, may play significant roles in determining tick abundance and diversity. Although we were not able to molecularly analyze the ticks collected from livestock, which would have been useful to confirm tick identities, the morphological identification revealed presence of *Hyalomma* species, recognized as a primary vector of CCHF [66,69]. CCHFV has also been detected in the other genera of ticks found during our study (*Amblyomma* and *Rhipicephalus*) [6], but their contribution to virus transmission remains less well established compared with *Hyalomma* spp. In surrounding countries of Burkina Faso, CCHFV was found in tick samples from the same three main genera in Ivory Coast and Ghana [38,70,71].

Furthermore, environmental and climatic factors contribute to the distribution of disease. In our study, aridity, mean NDVI, temperature, and precipitation were associated with cattle seroprevalence in univariate analyses. We observed significant regional differences in seroprevalence among cattle and small ruminants, with the more humid region (Hauts-Bassins) showing higher seroprevalence for both groups. In multivariable analysis, slope remained significantly associated with CCHF prevalence in cattle as for other similar studies [72,73]. These reconfirm that CCHF presence and risk is driven by ecological and climatic factors [74–77]. The ecological conditions of the savanna, arid and semi-arid land of Burkina Faso are ideal for *Hyalomma* spp. tick survival and growth with seasonal variations [25,69,78].

The null two-level mixed-effects logistic regression model with a random intercept for household for both cattle and small ruminants provides evidence that seroprevalence is not randomly distributed among animals but is instead clustered within households. The high ICC values highlight the importance of household-level factors in shaping exposure risk. Such clustering is expected in zoonotic systems where exposure to vectors (ticks) or infection sources (infected animals, shared pastures, and housing practices) is shared within households. Household-level clustering of vector-borne or zoonotic infections in livestock has been reported in other studies [56]. This suggests that shared environmental, ecological, and behavioral risk factors within households or herds play a central role in transmission dynamics [5,59]. In our study, the household-level factors identified include the presence of tick-infested animals, shared grazing with external herds, and risky handling or slaughtering practices. Notably, the higher ICC observed in small ruminants may reflect more homogeneous husbandry conditions within households compared to cattle, which may have more variable exposure due to grazing or movement patterns.

These findings have important implications for disease surveillance and control. They suggest that household-based interventions, rather than individual animal or human, could improve the efficiency of control programs [65]. For example, coordinated tick control, biosafety training, and improvement of animal housing at the household scale may yield higher impact than animal-level interventions alone [52].

While the study provided a set of informative data, some limitations encountered can be discussed. First, relying on seropositivity data without confirmatory testing using neutralisation tests may lead to false positives due to cross-reactions. Cross reactions with orthonairoviruses have been described in previous studies, where antibodies against related viruses can produce false positive results in ELISA-based assays [79,80]. This could affect the accuracy of the seroprevalence estimates for CCHF. Moreover, our targeted sampling approach may limit generalizability to non-farming populations or areas with different agro-ecological characteristics. The observed seroprevalence estimates should be interpreted within this context and may not represent a strict upper bound of national prevalence for non-farming populations. Second, the cross-sectional study design limits our ability to determine causal relationships between predictors and outcomes, as it does not account for temporal effects. Additionally, the reliance on self-reported data from questionnaires introduces potential biases, as these are less robust than observational data. Third, seasonal variations in tick activity and CCHF transmission may also have influenced the observed prevalence, as the study was conducted during the dry season (February and March). Finally, ecological and climatic variables were derived from secondary data and may not fully reflect micro-level exposures.

## Conclusion

Our study provides evidence of CCHF virus circulation among both humans and domestic animals in rural mixed crop-livestock farming systems in Burkina Faso. The identification of risk factors at both the individual and household levels offers insights into better-designed public health interventions, taking into consideration the One Health approach to improve coordination, for example, between communities, medical and veterinary services. The observed clustering of seropositive cases within households highlights the need to prioritise surveillance and prevention strategies among mixed crop-livestock farmers. The association between animal-level risk factors such as tick infestation and seropositivity supports the implementation of acaricide treatments to reduce transmission and improve livestock health. Risky biosafety practices identified also indicate actionable points for community sensitisation and improved amateur slaughtering conditions. Furthermore, the influence of agroecological variables on seroprevalence suggests that climate change may shift the geographic distribution of CCHF risk, requiring expanded surveillance in currently unaffected areas. Lastly, the detection of asymptomatic seropositive individuals underscores the importance of strengthening diagnostic and epidemiological capacity for undiagnosed febrile illnesses in rural settings in Burkina Faso and Sub-Saharan Africa.

## Supporting information

S1 Appendix Output of the null two-effect model with random intercepts and fixed effects of the cattle seropositivity to CCFH. (DOCX)

S2 Appendix Output of the null two-effect model with random intercepts and fixed effects of the small ruminants seropositivity to CCFH. (DOCX)

S3 Appendix Specification tests for Models. (DOCX)

S4 Appendix Databases used for analysis. (XLSX)
