# Supplementary material for: Sero-epidemiology of Crimean-Congo haemorrhagic fever in mixed crop-livestock farming households in Burkina Faso: a one health study
Source: PLoS One. 2026 May 4;21(5):e0347146. doi: 10.1371/journal.pone.0347146 (PMC13138657; doi:10.1371/journal.pone.0347146)
Supplement: S1 Appendix — (DOCX) [file pone.0347146.s001.docx]

**S1 Appendix 1:** Output of the null two-effect model with random intercepts and fixed effects of the cattle seropositivity to CCFH


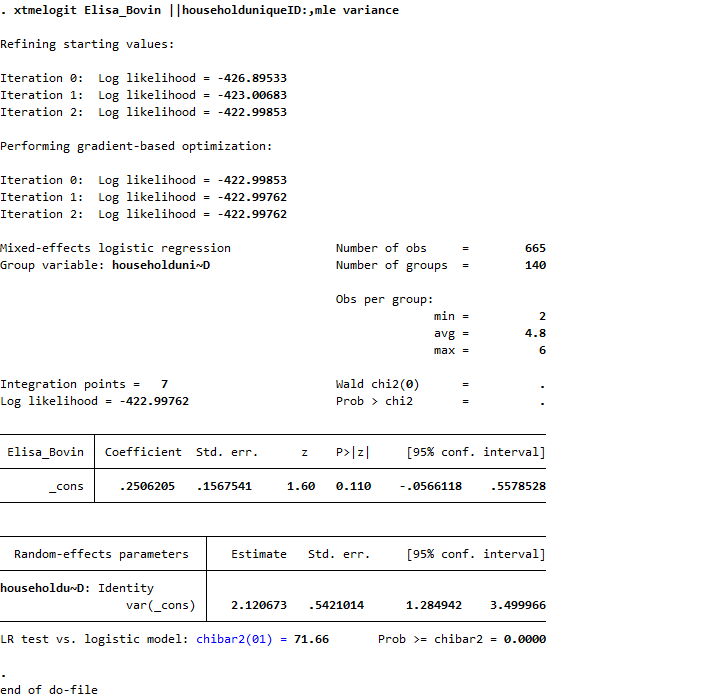


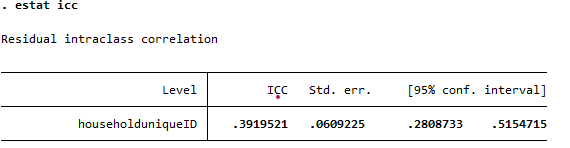


***Interpretation*** *The test statistic is 71.66 with a corresponding p-value of less than 0.05 (<0.001) and so there is strong evidence that the between-household variance for CCHF seroprevalence among cattle is non-zero. The Intra-cluster correlation (ICC) is 0.39 indicating that 39.2% of the total variance in cattle seropositivity is attributable to differences between households, rather than between individual animals*

**Figure S1:** STATA Output of the null two-effect model with random intercepts and fixed effects of the cattle seropositivity to CCHF analysis and ICC calculation
