# Supplementary material for: Sero-epidemiology of Crimean-Congo haemorrhagic fever in mixed crop-livestock farming households in Burkina Faso: a one health study
Source: PLoS One. 2026 May 4;21(5):e0347146. doi: 10.1371/journal.pone.0347146 (PMC13138657; doi:10.1371/journal.pone.0347146)
Supplement: S2 Appendix — (DOCX) [file pone.0347146.s002.docx]

**S2 Appendix :** Output of the null two-effect model with random intercepts and fixed effects of the small ruminants seropositivity to CCFH


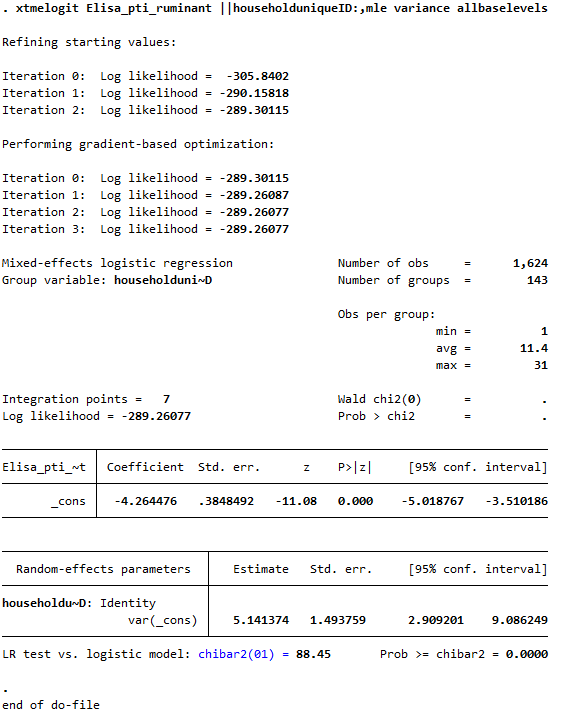


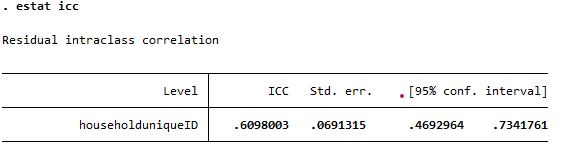


***Interpretation*** *The test statistic is 88.45 with a corresponding p-value of less than 0.05 (<0.001) and so there is strong evidence that the between household variance for CCHF seroprevalence among small ruminants is non-zero. The Intra-cluster correlation (ICC) is 0.61 indicating that 61% of the total variance in small ruminant seropositivity is attributable to differences between households, rather than between individual animals*

**Figure S2:** STATA Output of the null two-effect model with random intercepts and fixed effects of the small ruminants seropositivity to CCHF analysis and ICC calculation
