# Supplementary material for: Sero-epidemiology of Crimean-Congo haemorrhagic fever in mixed crop-livestock farming households in Burkina Faso: a one health study
Source: PLoS One. 2026 May 4;21(5):e0347146. doi: 10.1371/journal.pone.0347146 (PMC13138657; doi:10.1371/journal.pone.0347146)
Supplement: S3 Appendix — (DOCX) [file pone.0347146.s003.docx]

**S3 Appendix :** Specification tests for Models

**Figure S 3:** Graphic of the area under the Received operating procedure (ROC) curve of the residual of the multilevel modeling of the “cattle seroprevalence”

***Interpretation:*** *the postestimation test of the multilevel modeling of the “Cattle seroprevalence” show an area under the curve (AUC) value at 0.9085 which indicates excellent discrimination between the two outcome classes (positive vs. negative). This very good AUC suggests a high goodness of fit of the model.*

**Figure S 4:** Graphic of the area under the Received operating procedure (ROC) curve of the residual of the multilevel modeling of the “small ruminant seroprevalence”

***Interpretation:*** *the postestimation test of the multilevel modeling of the “small ruminant seroprevalence” show an area under the curve (AUC) value at 0.9284 which indicates excellent discrimination between the two outcome classes (positive vs. negative). This very good AUC suggests a high goodness of fit of the model.*
